# Developing the agenda for European Union collaboration on non-communicable diseases research in Sub-Saharan Africa

**DOI:** 10.1186/1478-4505-8-13

**Published:** 2010-05-19

**Authors:** Mark McCarthy, Dermot Maher, Adama Ly, Agbor Ndip

**Affiliations:** 1 UCL Department of Epidemiology and Public Health, University College London, 1-19 Torrington Place, London WC1E 6BT, UK; 2 MRC/UVRI Uganda Research Unit on AIDS, PO Box 49, Entebbe, Uganda; 3 André Wolff Institute, University Paris XI and AFROCANCER A.C. BP 60721, 75827 Paris Cedex 17 France; 4 Manchester Diabetes Centre, University of Manchester, 193 Hathersage Road, M13 0JE, Manchester, UK

## Abstract

**Background:**

Health research is increasing in Africa, but most resources are currently chanelled towards infectious diseases and health system development. While infectious diseases remain a heavy burden for some African countries, non-communicable diseases (NCDs) account for more than half of all deaths globally and WHO predicts 27% increase in NCDs in Africa over the next decade. We present findings of a European-Africa consultation on the research agenda for NCDs.

**Methods:**

A workshop was held in Yaoundé, Cameroon, organized by the Network for the Coordination and Advancement of Sub-Saharan Africa-European Union Science and Technology Cooperation (CAAST-Net). Drawing on initial presentations, a small expert group from academic, clinical, public-health and administrative positions considered research needs in Africa for cardiovascular disease, cancer and diabetes.

**Results:**

Research in Africa can draw from different environmental and genetic characteristics to understand the causes of the disease, while economic and social factors are important in developing relevant strategies for prevention and treatment. The suggested research needs include better methods for description and recording, clinical studies, understanding cultural impacts, prevention strategies, and the integrated organisation of care. Specific fields proposed for research are listed.

**Conclusions:**

Our paper contributes to transparency in the process of priority-setting for health research in Africa. Although the European Union Seventh Framework Research Programme prioritises biomedical and clinical research, research for Africa should also address broader social and cultural research and intervention research for greatest impact. Research policy leaders in Africa must engage national governments and international agencies as well as service providers and research communities. None can act effectively alone. Bringing together the different stakeholders, and feeding the results through to the European Union research programme is a valuable contribution of CAAST-Net.

## Introduction

The workshop to develop research priorities on non-communicable diseases (NCDs) in Africa was held in Yaoundé, Cameroon, 4-5 November 2009. It was organized by the Network for the Coordination and Advancement of Sub-Saharan Africa-European Union Science and Technology Cooperation (CAAST-Net) and hosted by the Institut de Recherche pour le Développement (IRD). This paper describes the process and findings of the workshop as a contribution to research development in Africa and for transparency in European policy-making.

### Developing research cooperation

CAAST-Net was established with funding through the European Union's Seventh Research Framework Programme (FP7) to advance research cooperation between Africa and Europe[1]. The four-year project, which started in 2008, supports Africa-Europe dialogue across a broad range of policy areas related to science and technology, and includes the including identification of specific research topics for support under the research Framework ProgrammeResearch FP7. It forms one of a group of projects advancing cooperation between Europe and Africa supported by the EU and is led by the Association of Commonwealth Universities on behalf of the International Science and Innovation Unit of the UK government. CAAST-Net maps African research issues of mutual interest for Europe, seeks a consensual approach from participants, and addresses broad themes for subsequent elaboration. The three main criteria to prioritise choices are: 1) enabling conditions (that capacity has to exist); 2) strategic imperatives - international policy support, for example in The New Partnership for Africa's Development (NEPAD) [2]; and 3) mutual benefit for Europe and Africa. In May 2009, the first panel considered research on energy, while later seminars address urban transport and social sciences research. Outputs from each seminar are specific researchable topics, while the broader outcome is strategic research cooperation between the two continents.

### Health research in Africa

Developments over the past decade have raised the profile of health research in Africa. The Abuja Declaration and Plan of Action from the African Summit on Roll Back Malaria in 2000 catalysed concern for both interventions and research for health. NEPAD, created in 2001 under the auspices of the Organisation of African Unity, recognises the contribution of health in supporting economic development in Africa. The Joint EU-Africa Strategy, adopted at the Lisbon EU-Africa Summit in 2007, comprises eight thematic partnerships, including one for Science, Information Society and Space. The Global Ministerial Forum on Research for Health in Bamako, Mali in 2008 affirmed the importance for Africa of health research, and particularly for research on non-communicable diseases [3].

### Non-communicable diseases in Africa

The epidemiological transition and resulting rise in non-communicable diseases gives prominence to non-communicable diseases (including cardiovascular disease - heart disease and stroke - diabetes and some cancers), which together now account worldwide for up to 60% of deaths, and almost half of all 'premature' deaths [4]. WHO predicts that NCD deaths globally will increase by 17% over the next decade, with the greatest increase in the African region (27%) [5]. Yet in sub-Saharan Africa, governments, donors and research-funders have channelled most resources into infectious diseases, especially the trio of HIV, malaria and tuberculosis; and initiatives such as the European and Developing Countries Clinical Trials Partnership (EDCTP) have been focused on treatment and healthcare services for these infections rather than the needs and opportunities for interventions against NCDs, through clinical trials, changing lifestyles and public policy.

Recent favourable developments in support of addressing NCDs include the WHO Global Strategy for the Prevention and Control of Noncommunicable Diseases [5] and the establishment by WHO, the World Bank, the World Economic Forum, and three international NGOs (the World Heart Federation, International Diabetes Federation and the International Union against Cancer), of a global NCDs network (NCDnet) to spread initiatives on NCDs [6]. The network will join health-care and preventive approaches, and also enable research on interventions at primary care level [7].

### Determining NCD research priorities

There is a limited literature on non-communicable disease research priorities in developing countries. Unwin et al [8] reported conclusions of 'discussions over several years' between clinicians in UK and two African countries. They recommended clinical research in three broad areas - surveillance, treatment and prevention - without a basic sciences biomedical approach. Daar et al [4] drew on views from experts and representatives from international organisations and research agencies. Publishing in Nature, they proposed 39 topics for research across six broad socio-political areas: raising public awareness, enhancing policies, modifying risk factors, engaging business and communities, mitigating impacts of poverty and urbanisation, and reorienting health systems. Ebrahim and Smeeth [9] summarised existing literature on NCDs relevant to low and middle-income countries, but also recommended that research within individual countries, rather than simply drawing from world scientific literature, is crucial to changing government agendas and policies.

### European Union health research programme

The European Union Seventh Research Framework Programme runs from 2007 to 2013. It has four broad sub-programmes: Cooperation (thematic research); Ideas (researcher-driven proposals); Capacities (supporting structures); and Marie Curie (supporting individuals). Health is the second best-resourced of the thematic calls within the Capacities programme, distributing around seven hundred million Euros a year. Calls for research are made annually. As well as researchers within the EU, and the 'associated states' which also contribute financially to the programme, research collaboration is encouraged with countries across the world. Some parts of the call are focused on international cooperation with specific geographical regions and countries beyond the EU. For the first time in 2009, topics for cooperation with Africa in three thematic areas - health, food and environment - were launched as a coordinated joint call. Further calls are anticipated for the period 2010-2013.

## Methods

The CAAST-net consultation brought together a small group of experts, selected from African and European countries and with a range of disciplines, for a two-day meeting in Yaoundé, Cameroon during 4-5 November 2009. Prior to the consultation, experts and stakeholders were asked to submit short abstracts on potential research issues, and 17 of these were received and considered within the consultation. The objective of the meeting was to recommend broad areas for research for the three NCDs (cardiovascular disease, cancer and diabetes) identified as having particular relevance for Africa-Europe collaboration, and for integrated approaches to these NCDs. The process of consultation in the workshop did not seek to be exhaustive of knowledge and research initiatives on the topics. Consideration of the specific proposals submitted in advance served to initiate discussion, resulting in the identification of broad topics to be considered and further elaborated for inclusion in further calls under the health theme. This report records the suggestions and discussions of the group over the two days. We have added references which record the background information rather than a full literature review.

## Results

Research can use the different environmental and genetic characteristics present in Africa to better understand the causes of the disease, and help develop strategies relevant for prevention and treatment.

### Cardiovascular disease

African aspects of cardiovascular diseases were considered to include the wide population distribution of hypertension, and impacts including premature stroke, heart and renal failure. The burden of cardiovascular disease in Africa is growing, and contributing conditions, such as diabetes and obesity, are also increasing rapidly. There is an urgent need for more comprehensive and representative data to define better this burden across Africa and for studies on disease control.

While known risk factors have an important place, there is no equivalent of the Framingham longitudinal study providing data about populations and factors of disease progression for Africa. Areas of further investigation could include salt sensitivity and arterial stiffness - which may have genetic components. Since malnutrition rather than dietary excess continues to be a pressing problem in rural Africa, low birth weight and sub-optimal development may be an important determinant of cardiovascular diseases in later age for people moving to towns and cities and taking up western lifestyles.

Cultural, ethnographic and geographical analyses are important, since there may be differences between western and African concepts of health and disease. Cultural attitudes towards obesity, for example, may have marked differences within and between continents, such that in some societies obesity may be seen as a sign of wealth or a feature of beauty.

There is a lack of representative information within countries, and insufficient information on differences between countries. There is a need for the development of structures for gathering information, networks to share data and support analysis, and better use of information to provide health policy makers with the evidence base for developing and adopting new approaches. At local level, systems developed for HIV treatment and monitoring could form the basis for the development of shared disease and treatment registries. These data, when linked to differences in cultures, are also of great interest to social scientists.

Areas of concern include how to organise the health system to provide an effective response to the challenges posed by cardiovascular disease, the evaluation of cost-effectiveness of different means of care delivery, and the provision of the related social support programmes needed. Developments need to be adapted to the population, including for example innovations in diagnostic tests, outreach support through tele-medicine and improved access to medicaments, with the value determined by cost-effectiveness studies.

The discussion indicated the following research topics:

Studies assessing the effectiveness of prevention, e.g. promoting health and changing lifestyles

Studies explaining variations in disease incidence - assessing the role of risk factors (e.g. low birth weight), genomics, and cultural aspects

Studies aimed at improving data on incidence and outcomes of treatment and care

Developing networks for data collection, sharing and analysis

Studies of how varying cultures mediate in treatment effectiveness

Comparing disease risk and occurrence in Africans in Africa and Africans who are migrants to Europe

Studies of ways of improving diagnosis, including ways of providing advice at a distance

### Cancer

While WHO's leadership has helped to increase attention to cancer worldwide, there has been significant progress in raising the profile of cancer in Africa, including the systematic description of what is currently known [10]. In addition to cancers which are common in both Europe and Africa, e.g. breast, colon, lung and prostate, there are significant impacts in Africa of cancers strongly associated with virus infection, e.g. cancer of cervix and Kaposi sarcoma, and concern for skin cancer in albinos.

CARISA [11] reported the 'landscape' of cancer research of South Africa. Cancer research support includes data management, diagnostics (biomarkers, genomic profiling, imaging techniques), prevention, treatment, and palliation and social aspects. But their main areas proposed for research included molecular carcinogenesis, genetic and molecular epidemiology, and therapeutic development.

However, while there is much interest in the links between genetics and cancer, Africa has a diverse gene pool, limiting the feasibility of epidemiological studies. Moreover, as yet no-one has identified human protein markers, and proteomics is beyond the financial resources of most African countries.

There is an urgent need to improve diagnostic services, including the evaluation of diagnostic innovations such 'telepathology' (as there are very few pathologists in Africa) and to improve treatment services, including the provision of common anti-cancer drugs and morphine for palliation.

Cultural factors for investigation include the possible protective effects of diet against cancer (e.g. drinking Rooibos tea in South Africa) and plants (e.g. maize as a factor in the lower prevalence of colon cancer among black than among white South Africans).

Knowledge is needed to support ministries of health in developing evidence-based policies for the implementation of interventions which are widely available or are being widely introduced in Europe but are not yet available in Africa. For example, cost-benefit and health politics studies are needed on the impact of human papilloma virus vaccination and cervical cancer screening programmes on cervical cancer prevention. Public demand also needs consideration.

The discussion indicated the following research topics:

Studies of aetiology, e.g. epidemiological investigation of variations in incidence of common cancers, differences of environment, viruses, lifestyle (including diets), and genetic markers

Studies on the effective organisation of services, including improved diagnosis (e.g. telepathology for identification of lymphoma) and treatment (e.g. cost-effectiveness and equity of access in provision of chemotherapy and radiotherapy), and palliative care (e.g. pain control)

Occupational cancers - studies of ethical and legal issues, protection of workers, mines, issues of plantations

Scoping study of emerging diagnostics, including proteomics

Studies of better data collection, including cancer registration, management and assessment of impact

Effectiveness and cost-effectiveness of prevention strategies: vaccines, tobacco, diet, alcohol, information and education for lifestyle change. Policy research to inform decision-makers choosing different prevention strategies

Psychological and cultural approaches to cancer as a disease, and towards end-of-life care.

Evidence for policy-makers to decide between different models of cancer, including assessment of equity of access and outcome

### Diabetes

There are estimated to be 12 million people with type 2 diabetes in Africa, and the numbers are growing by 1 million every two years. Type 2 diabetes in European countries is due to increases in obesity. Similarly, explanations for the rising prevalence of diabetes in sub-Saharan Africa concern obesity through lifestyle changes due to globalisation and urbanisation, although in Africa it is as yet the more affluent sections of society who are becoming more sedentary and overweight. One participant reported that a study in Cameroon showed a prevalence of type 2 diabetes of about 6%, with a prevalence of impaired glucose tolerance ('borderline diabetes") of up to 1 - 3%. More comprehensive and representative data in Africa are needed.

A critical issue for epidemiological studies is in defining standards for diagnosis. Reference values from western countries may not necessarily be the same for Africa. For diabetes there is debate whether HbA1_C _is superior to fasting blood sugar and glucose tolerance test, because of its relatively high cost and the potential difficulty in its interpretation in the presence of haemoglobinopathies and glucose-6-phosphate dehydrogenase (G6PD) deficiency. The suggestion that differentiation between type 1 and type 2 diabetes is less clear cut in Africa needs further study.

Research may investigate whether there are characteristics for people who develop diabetes which are specific to Africa, such as unique genetic polymorphisms and interactions with the environment (epigenetic phenomena). For example, in parts of Africa, such as around the great lakes, the soil contains much iron and there are clinical reports of unusual forms of diabetes in patients who are not obese and relatively young, which may be associated with pancreatic iron overload. Nutritional factors are important, including the 'double burden of malnutrition' - the relationship between malnutrition in maternal and early infancy periods and later dietary excess and lack of physical activity, leading to diabetes.

Optimal diabetes control and management can decrease the risk of end-organ damage. Research is needed on improving the primary care response to the needs of people with diabetes, including cost-effective ways of recognising organ damage and improving clinical management, and new approaches to complications such as diabetic foot disease. There are examples of a few preventive and care programmes, e.g. in certain sites in Nigeria and the Democratic Republic of the Congo, but these are in the private healthcare sector. Half a million people worldwide have chronic diabetic kidney disease. The numbers affected by renal disease in Africa and the financial implications for diagnosis and treatment need to be further explored.

The discussion indicated the following research topics:

Coordinated studies of prevalence and trends of diabetes in different settings.

Studies to evaluate implementation of a structured approach to diagnosis and treatment, with improved data collection on diagnosis and treatment outcomes to inform better service delivery.

Studies on improving access to diagnosis and care, with care delivery closer to the patient, e.g. in community settings and through enhanced role of non-medical staff.

Assessment of effectiveness of early detection of end-organ damage (for burden of disease and service planning, e.g. foot care problems)

Studies of dietary transition from rural malnutrition to different diets in urban settings, the role of maternal malnutrition, extent and mechanisms of insulin-resistance, and barriers and resilience of communities for prevention.

Biotechnical approaches to aetiology - genetics, resistance, blood markers, animal models.

Urbanisation - environments affecting human behaviours - causing diabetes and the potential for alternative urban settings

Studies on provision of affordable diagnosis, case-detection and screening

### Integrated approaches to NCDs

In addition to the research topics identified for each specific NCD, research is also relevant to the NCDs collectively, since they share common risk factors, interactions, and similarities in approaches to prevention and care.

Common risk factors including such as smoking, obesity, alcohol and hypertension contribute to cardiovascular disease and its complications, as well as cancer and diabetes. Integrated delivery of health services for prevention and care, as well as multisectoral policies for prevention, can contribute to reduction of all of these diseases.

Since important social and cultural perspectives may be relevant across different NCDs, studies of prevention and clinical management should address NCDs as a group. This can be more efficient, avoiding duplication of effort: for example, developing a standard approach to registration and follow-up of patients, and developing policies for prevention based on shared risk factors. The cost of pharmaceuticals, especially in long-term care, imposes a serious burden on African countries, and competes in budget with other health priorities including finance for research.

The discussion indicated the following research topics:

Measurement of prevalence and trends over time of common shared risk factors of NCDs in different settings in Africa, e.g. urban, semi-urban and rural

Studies of management of NCDs in primary care settings, including records systems, diagnosis and management protocols, and drug procurement

Studies on policies and practice for lifestyle changes to meet shared risk factors in NCDs, including physical activity, smoking, improved diet and decreased alcohol intake

Studies on health systems and quality of care delivery, e.g. human resources, financing, procurement of drugs and supplies, and organisation of health facilities

## Discussion

Research on non-communicable diseases in Africa is 'a priority, not a distraction' [9]. While malnutrition and famine remain the predominant concerns in rural areas, and infectious diseases continue to impose an heavy buruden in some African countries, NCDs are emerging as the major health threat of middle-income countries and are increasingly important in urban areas of even the poorest countries. Just as control of infectious diseases through environmental means assists the whole population, so also action on behavioural disease determinants - smoking, diet, physical activity - will also assist broad sections of the population. It may be argued that the determinants of late-onset diabetes are very similar to those for cardiovascular disease, while a third major field of morbidity and mortality should be considered for prevention research - road accident trauma, which is rising rapidly as mobility in Africa increases. But research linking transport and health is not a priority area for the European Union, nor are these topics as yet prioritised together for Africa.

The workshop noted biological and laboratory issues, clinical and epidemiological features, and also research on the organisation of prevention and care, reflecting the broad backgrounds of the participating panel. The process did not seek to be exhaustive of knowledge and research initiatives on the topics, but rather to extend broadly and indicate topics for calls whereby more focused areas can be proposed. One referee for this paper, however, noted a lack of interventional topics - "ie trying out new types of intervention at public health or clinical levels, and how such intervention/treatments should be organised": these could include initial pilot studies, outcome clinical trials and Ministry-level projects as experimental interventions. There is also a strong rationale for considering the NCDs collectively, since they share common risk factors and common interactions, i.e. between obesity, smoking and exercise, and diabetes, hypertension and cardiovascular disease. The delivery of health service interventions for prevention and care, and the often multisectoral policies aimed at preventive measures, contribute to a reduction of all of these diseases [5]. It is therefore necessary to consider not only research topics relevant to specific NCDs, but also research topics which represent a joint approach to the different NCDs together.

Funding for research through the EU Research Framework Programme has grown rapidly in the past decade, because policymakers have urged a link between investment in research, innovation and economic improvement. Health research has also risen in priority, competing with agricultural, transport and IT research. The Seventh Research Framework Programme has encouraged participation of researchers across the world as partners in collaborative projects and programmes, and the 2010 calls for research related to Africa in three fields - health, agriculture and transport - were presented together with a total budget of over €20 m (although evaluated within the existing disciplines). The annual calls in FP7 are usually for 3-year proposals by a consortium of partners in both Europe and Africa, with total budgets around €3 m. CAAST-Net, itself funded through a competitive call in FP7, provides a mechanism for researchers to share their interests, to suggest priorities to the programme, and to develop collaborations. This present paper, reporting a CAAST-Net meeting, seeks to add transparency to this process.

Specific recommendations for research themes at the workshop were consolidated, developed into potential call texts, and distributed for further consultation before submission to the European Commission. The European research framework programme has a multitude of inputs, and an annual cycle which iterates between external advice and internal programme development. European member states have a strong influence through the programme committees, although in the health field national research councils give more support to laboratory and biomedical topics than to public-health research [12]. Cooperation between European and African partners is possible under the EU research framework programme across a range of topics of mutual interest and benefit.

While as yet the European Union Research Framework Programme prioritises biomedical and clinical research, research for Africa should address broader social and cultural research for greatest impact. Research on non-communicable diseases in Africa can contribute both to African development and also to better understanding of these diseases. Policy responses to NCD disease research in Africa must engage national governments and international agencies as well as service providers and research communities. None can act effectively alone,. Bringing together the different stakeholders was a valuable contribution of CAAST-Net, and the workshop proposals will be among inputs to inform calls for joint European-African health research in the coming years.

## Competing interests

The authors declare that they have no competing interests.

## Authors' contributions

All authors participated in the Yaoundé workshop. MM conceived and drafted the paper, DM, AL and AN provided expert commentaries and corrections. All authors read and approved the final manuscript.

## References

[B1] CAAST-Net http://www.caast-net.org/xwiki/bin/view/Main/

[B2] NEPAD (New Partnership for Africa's Development) http://www.nepad.org/home/lang/en

[B3] Stuckler D King L Robinson H McKee M WHO's budgetary allocations and burden of disease: a comparative analysis Lancet 2008 372 1563 1569 10.1016/S0140-6736(08)61656-6 18984189 PMC7159087

[B4] Daar AS Singer PA Persad DL Pramming SK Matthews DR Beaglehole R Bernstein A Borysiewicz LK Colagiuri S Ganguly N Glass RI Finegood DT Koplan J Nabel EG Sarna G Sarrafzadegan N Smith R Yach D Bell J Grand challenges in chronic non-communicable diseases Nature 2007 450 7169 494 6 10.1038/450494a 18033288

[B5] World Health Organization 2008-2013 Action Plan for the Global Strategy for the Prevention and Control of Noncommunicable Diseases 2008 Geneva, Switzerland: World Health Organisation

[B6] World Health Organisation Report on the progress made in implementing the 2008-2013 Action Plan for the Global Strategy for the Prevention and Control of Noncommunicable Diseases (June 2008 - February 2010). Background Paper http://www.who.int/ncdnet/events/background_progress_20100224.pdf

[B7] Maher D Sekajugo J Harries AD Grosskurth H Research needs for an improved primary care response to chronic non-communicable diseases in Africa Trop Med Int Health 2009 15 2 175 181 10.1111/j.1365-3156.2009.02438.x 20002618

[B8] Unwin N Seal P Rashid S Mugusi F Mbanya J-C Kitange H Hayes L Edwards R Aspray T Alberti KGMM Non-communicable diseases in sub-Saharan Africa: where do they feature in the health research agenda? Bull World Health Organ 2001 79 947 953 11693977 PMC2566676

[B9] Ebrahim S Smeeth L Non-communicable diseases in low and middle-income countries: a priority of a distraction? Int J Epidemiol 2005 34 961 966 10.1093/ije/dyi188 16150869

[B10] Ly A Khayat D Eds About cancer in Africa: from epidemiology to biomedical research applications and perspectives 2006 Paris: National Cancer Institute

[B11] Thiel L Roberts K Situational/landscape analys is of South African and international cancer research 2008 Cape Town, South Africa CARISA (Cancer Research Initiative of South Africa)

[B12] Conceição C Leandro A McCarthy M National support to public health research: a survey of European ministries BMC Public Health 2009 9 203 10.1186/1471-2458-9-203 19555492 PMC2713230

